# A Qualitative Study of Motivators, Strategies, Barriers, and Learning Needs Related to Healthy Cooking during Pregnancy

**DOI:** 10.3390/nu13072395

**Published:** 2021-07-13

**Authors:** Travertine Garcia, Kerith Duncanson, Vanessa A. Shrewsbury, Julia A. Wolfson

**Affiliations:** 1 Department of Nutritional Sciences, University of Michigan School of Public Health, Ann Arbor, MI 48109, USA; travi@vt.edu; 2 Department of Human Nutrition, Foods & Exercise, Virginia Tech, Blacksburg, VA 24061, USA; 3 Priority Research Centre for Physical Activity and Nutrition, The University of Newcastle, Callaghan 2308, Australia; kerith.duncanson@newcastle.edu.au (K.D.); vanessa.shrewsbury@newcastle.edu.au (V.A.S.); 4 School of Health Sciences, College of Health, Medicine and Wellbeing, The University of Newcastle, Callaghan 2308, Australia; 5 Department of International Health, Johns Hopkins Bloomberg School of Public Health, Baltimore, MD 21205, USA; 6 Department of Health Management and Policy, University of Michigan School of Public Health, Ann Arbor, MI 48109, USA

**Keywords:** pregnancy, culinary nutrition, cooking skills, diet quality, food skills, cooking education, qualitative study

## Abstract

Nutrition during pregnancy has lifelong impacts on the health of mother and child. However, this life stage presents unique challenges to healthy cooking and eating. Cooking interventions show promising results, but often lack theoretical basis and rigorous evaluation. The objective of this formative, qualitative study was to explore motivators, strategies, and barriers related to healthy cooking during pregnancy. Pregnant individuals’ preferences for a cooking education program were also explored. We conducted five focus groups with pregnant individuals (*n* = 20) in Southeast Michigan in 2019. Focus groups were audio-recorded and transcribed verbatim, then double coded by two members of the research team. Mean gestational age was 18.3 ± 9.6 weeks. Common motivators included feeding other children, avoiding pregnancy complications, promoting fetal growth, and avoiding foodborne illness. Challenges included pregnancy symptoms, navigating nutrition recommendations, mental energy of meal planning, family preferences, and time constraints. Strategies employed were meal planning and including a variety of foods. Participants identified organizational strategies, recipes, nutrition information, and peer support as important components of a cooking intervention during pregnancy. This study characterized multiple challenges to healthy home cooking during pregnancy, providing novel insight to inform the development of cooking skills education programs during this important life stage.

## 1. Introduction

Nutritional status during pre-conception, pregnancy, and postpartum is associated with long-term health outcomes for both mother and child [1,2,3]. Maternal obesity rates range from 24% to 35% in high-income countries and have increased substantially since the 1970s, as has the incidence of excessive gestational weight gain [4]. Overweight and obesity are key risk factors for gestational diabetes, hypertensive disorders of pregnancy, and excessive gestational weight gain [5,6,7]. These conditions are associated with increased risk of maternal, perinatal, and neonatal complications including early pregnancy loss, abnormal fetal growth patterns, labor complications, preterm birth, postpartum weight retention, childhood obesity, and cardiovascular disease and impaired glucose metabolism in mother and child [4,6]. Additionally, the first 1000 days of life—from conception to age two—have been identified as a critical period for physical and cognitive development during which optimal nutrition is key [1,8,9]. Thus, interventions to improve diet quality during pregnancy provide a unique and powerful opportunity to improve health across the lifespan.

Diet quality is a key contributor to maternal obesity and excessive gestational weight gain, and an independent risk factor for gestational diabetes [4,6]. In high-income countries, diet quality is generally poor, with consumption of vegetables and fruits far below national guidelines and consumption of foods high in saturated fat, sodium and sugar far above national guidelines [10,11,12,13]. Trends in diet quality have been influenced in part by decreased home cooking and increased consumption of convenience foods, which tend to be higher in calories, fat, sodium and sugar [14,15,16]. More frequent consumption of home-cooked meals is associated with higher diet quality, and therefore many interventions have targeted home food preparation as a key health behavior for weight management and obesity prevention in the general population [17,18,19]. However, pregnancy presents many unique challenges to cooking and eating healthy meals, such as physical symptoms, competing priorities, and family/peer pressure with regards to food choices [20,21]. Pregnancy is therefore a key life stage to encourage and support healthy cooking and eating.

Many nutrition-focused programs and policies already target pregnant women and their families, with the aim of facilitating improved diet quality and health for mother and child [22,23]. However, relatively few have used cooking skills education as a key strategy for improving diet quality [14]. Cooking skills interventions have been shown to result in short-term improvements in cooking skills, nutrition knowledge, diet quality, infant feeding practices, and parent and child health outcomes [14,24]. However, very few cooking interventions target individuals during pre-conception or pregnancy, compared to postpartum [14,24], despite the well-established need for dietary interventions during these life stages [2,4,23]. Furthermore, cooking skills interventions often lack theoretical basis and rigorous evaluation; they are often delivered as one component of a lifestyle intervention and rarely examine clinical outcomes, and lack formative research to inform program development [14,25,26,27].

The objective of this study was to explore barriers, motivators, and strategies related to home cooking among a sample of pregnant women in Southeast Michigan, USA, as well as to understand what pregnant women would like to see in a cooking education program. The goal of this research is to inform the development of future cooking skills interventions to address the specific needs of pregnant women and their families.

## 2. Materials and Methods

### 2.1. Study Design

We conducted a formative qualitative study using focus groups to characterize cooking behavior during pregnancy and elicit information that could inform the development of a cooking education program for pregnant women. The consolidated criteria for reporting qualitative research (COREQ) checklist for interviews and focus groups was used to guide reporting. This study was approved by the University of Michigan Institutional Review Board (HUM0016622).

### 2.2. Focus Group Participant Recruitment

Recruitment strategies included an online participant recruitment platform at the University of Michigan, social media advertisements, and flyers posted at public locations including prenatal clinics, local Special Supplemental Program for Women Infants and Children (WIC) offices, cafes, libraries, grocery and other stores. Inclusion criteria were being at least 18 years old, using English as a primary language, and being currently pregnant (primigravida or multigravida). Additionally, in order to focus on women who were at higher risk for nutrition-related pregnancy risk factors, participants were required to meet one of the following criteria: having a family history of type-2 diabetes, having a first-degree relative (mother or sister) who has had gestational diabetes, or having a BMI ≥ 25 kg/m^2^. After indicating interest in this study via phone or email, each participant was screened for eligibility by phone by T.G. This study was described to participants over the phone and again when they arrived for focus groups; participants provided oral consent at that time.

### 2.3. Focus Group Procedures

Five focus groups were held in October and November of 2019 in private meeting rooms at public libraries or WIC offices in Southeast Michigan, USA. Focus groups were held on both weekends and on weekday evenings to accommodate scheduling preferences of the participants. Each participant attended only one focus group. Focus groups were approximately 90 min, were audio-recorded, and were moderated by T.G. The moderator was a female second-year public health nutrition graduate student with prior experience facilitating focus groups. A public health undergraduate student attended one focus group to observe. The moderator introduced herself at the beginning of each focus group and explained the purpose of the focus group to “learn about participants’ experiences around food, shopping, cooking and eating at this stage of life…to help develop a cooking and food skills program for pregnant women and potentially their partners.”

Participants completed a brief demographic survey as they arrived, prior to the start of the focus group. In the survey, participants were also asked “how many days in an average week do you, or someone in your household cook dinner?” and whether they had a working stove, oven, microwave, refrigerator, and/or freezer in their home. Light refreshments were provided and participants received a $30 gift card as compensation for their time.

A discussion guide (Appendix A) was developed by the research team specifically for this study. The focus group began with an ice breaker question, asking everyone to share their favorite dish to cook or eat, and a short discussion of general thoughts and attitudes about cooking. The first set of focus group questions focused on motivators and challenges to cooking during pregnancy, as well as anticipated challenges postpartum. The second set of questions elicited discussion about how cooking relates to health and how priorities around cooking, health, and food safety may change during pregnancy. The third set of questions focused on household cooking dynamics prior to and during pregnancy. The final set of questions explored what participants would like to see in a cooking program during pregnancy, including program content, timing, instructional format, and logistical considerations.

### 2.4. Data Analysis

Audio-recordings from the focus groups were transcribed verbatim by a professional service. All transcripts were double coded by two members of the research team (T.G. and J.A.W.) using a codebook developed a priori by the research team based on the focus group question guide. Coding proceeded iteratively, and transcripts were revisited as new codes were identified and added to the codebook [28]. Throughout coding, the research team met to discuss interpretation of codes. HyperResearch software was used to facilitate coding and analysis. After initial line by line coding was complete, codes were aggregated into broader themes, and code memos were produced for each theme and code grouping [29]. The research team determined that data saturation was reached and additional focus groups were not needed based on preliminary first-level analysis and coding by two members of the research team (T.G. and J.A.W.) [30]. Transcripts were not returned to participants for comment.

## 3. Results

### 3.1. Focus Group Participants

Among 31 eligible volunteers, 30 agreed to participate in focus groups and one declined due to lack of childcare. Ten participants cancelled or no-showed; three cited inconvenient location, one was too close to term, and the others did not give a reason. Twenty individuals participated in the focus groups. Characteristics of the focus group

Participants are presented in Table 1. The mean age of participants was 32.1 ± 5.6 years. Mean gestational age was 18.3 ± 9.6 weeks. The majority of participants were college graduates (76%), married or living with a partner (81%), and working part time or full time (81%). Two-thirds of participants had children already (67%) and reported that they or someone in their household cooks dinner five or more times per week (70%).

Over half (60%) of participants described themselves as overweight prior to their current pregnancy.

### 3.2. Focus Group Themes

Themes are discussed below with exemplar quotations; where relevant, differences in views between participants (e.g., women experiencing their first pregnancy vs. women with children) are noted. Main themes for each topic with key sub-themes are presented in Table 2.

#### 3.2.1. Motivators to Home Cooking during Pregnancy

Most participants enjoyed cooking and considered themselves to be good cooks; a minority of participants did not enjoy cooking, were not confident in their cooking abilities, or enjoyed baking (i.e., making cakes, cookies, etc.) but not cooking (i.e., preparing meals). Participants identified multiple motivators to cooking during pregnancy, including feeding their other children, avoiding pregnancy complications, promoting the growth of baby in utero, and avoiding foodborne illness. Home-cooked meals were perceived as safer and healthier than meals procured outside the home, with emphasis on knowing what ingredients are in foods and whether they are prepared safely, and avoiding excess salt, sugar, and fat.

*I know when I make foods, since I’m usually using the vegetable itself or the fruit itself, I’m taking it from the most raw form and then creating something with it, I know exactly what’s going into it and I feel like overall it ends up being healthier [and] I enjoy it more because I created it.*

*—Participant 15, age 36, mom of 1*

For some women—especially first-time moms and/or those with a history of infertility or pregnancy loss—promoting the health and growth of the fetus in utero made them more conscious about their food choices.

*Since getting pregnant I immediately started eating things high in calcium and fiber and things like that just to promote the growth of the baby. And things that are healthy for me, too.*

*—Participant 9, age 42, first-time mom*

Participants perceived nutrition to be equally if not more important during breastfeeding (for both mother and child) and thus felt that cooking was important postpartum, albeit more challenging.

However, for those with previous pregnancies, wanting to set a good example for their children was a primary motivator for cooking healthy meals, more so than pregnancy itself. Many participants with previous pregnancies reported that they were less motivated to cook during their first pregnancies when they did not have other children to feed. During subsequent pregnancies, women needed to prepare meals for their children and wanted those meals to be healthy, so they felt more obligated to cook.

*With this [pregnancy], though, because of having [another] little one it encourages you to cook more and have a little more variety. So this time I definitely try to get a full variety because I want [my toddler] to eat good, so I pretty much cook for her and then I eat whatever she eats. <laughs> So I think that helps having another creature to take care of.*

*—Participant 15, age 36, mom of 1*

Avoiding pregnancy complications was another important motivator to cooking. Common concerns included excessive weight gain, gestational diabetes, preeclampsia, foodborne illness, and miscarriage. Those who had experienced complications in previous pregnancies were less inclined to consume food prepared outside the home and more conscious of nutrition when cooking at home.

*I’m just aware of sodium. I don’t have high blood pressure normally but just knowing that that happened in my last pregnancy, like, not wanting to repeat that. Yeah. So I do cook more, I cook almost every day …just knowing that I can control the sodium if I’m making it*

*—Participant 16, age 33, mom of 1*

Those with pre-existing health conditions including obesity, polycystic ovary syndrome, and pre-diabetes and lupus were also more concerned about complications of pregnancy and tended to place greater value on cooking as a health behavior.

*I have lupus so it’s already hard because I’m super high risk. When I eat, I try to eat, like, ‘okay, this is going to be good for me. It’s going to be good for the baby. And it’s also going to help try to keep some of the underlying health problems I have kind of at bay.’*

*—Participant 20, age 23, first-time mom*

#### 3.2.2. Cooking Strategies and Household Dynamics

Regardless of whether they enjoyed cooking, most participants were the primary cooks in their households and were responsible for food provision even when they were not cooking, for example, planning meals, giving instructions to their spouse, or buying ready-to-eat meals. For most, household cooking dynamics had not changed significantly during pregnancy, though some reported getting more help from their partners or parents.

*Recently, with me being tired and everything, [my husband] is probably cooking five nights a week. So, we’ve kind of flipped, but I try to at least make sure and take something out of the freezer…So, it’s minimal work for him, but it’s just the fact that I can’t get myself to do it.*

*—Participant 13, age 34, mom of 2*

*I would say the dynamics haven’t really changed because. I work full-time from home so…it just makes sense for me to do the cooking logistically…But he hasn’t really realized the point of my mental exhaustion with that…He’s just like, “Well, just do it.” I’m like, “No. That’s not going to happen. I need to sit down now.” It hasn’t changed yet but I’m hoping that it will soon.*

*—Participant 10, age 30, first-time mom*

When they described cooking healthy meals, participants focused on incorporating vegetables, balancing different food groups (protein, starch, and vegetables), using less processed ingredients, and including a variety of different foods. For some, particularly those with a history of excessive weight gain, preeclampsia, or elevated blood glucose, limiting calories, sodium, and/or carbohydrate was also a priority when cooking at home. Some identified reading food labels when shopping as an important step to cooking healthy meals at home.

Meal planning was an essential organizational strategy for many participants. Whether they cooked daily or cooked larger quantities less frequently and used leftovers, planning ahead helped participants save time and avoid burnout. Some participants chose highly structured approaches, citing specific cookbooks and methods, while other described meal planning in more general terms.

*I mean if I had more time I would probably prepare meals on the daily but because of my busy schedule I do a lot of meal prep on the weekend and then like two or three dishes. I’ll do a pulled pork and then that pulled pork translates into tacos one night or into a pasta dish on another night. So I try to be efficient with what I cook on the weekend just so we have something fresh.*

*—Participant 9, age 42, first-time mom*

Buying ingredients in bulk and freezing was another popular strategy, especially for meats, fish, and poultry, to ensure that healthy ingredients were always on hand.

*I sort of want to keep the balance of protein and veggie and fruit… we buy a lot at one time…and then I separate into small bags. So I think one small bag per day and then every morning “What kind of meal should we plan for the next day?” And we take it out and then we know what kind of meat we eat that day or fish or chicken and then what kind of veggies go with it.*

*—Participant 14, age 36, mom of 2*

#### 3.2.3. Barriers to Cooking during Pregnancy

Despite strong intentions around food and nutrition, participants faced multiple barriers to maintaining a healthy diet and cooking routine.

*When I first got pregnant I was like, yeah, I’m going to eat so healthy! I am super motivated to feed the baby healthy things and keep my body full of nutritious foods! But I had to give up on that because I could not stomach anything…thankfully it’s better now in the second trimester, but now I’m just really hungry and I feel like nothing I eat keeps me full.*

*—Participant 10, age 30, first-time mom*

Physiological symptoms of pregnancy, including aversions, nausea, fatigue, and cravings made cooking more challenging, especially during the first trimester. For many, nausea made it difficult to tolerate the smell of cooking and aversions limited the variety of acceptable ingredients. This made meal planning more difficult, if not impossible, since participants could not rely on being able to cook or eat what they had planned.

*I guess it kind of goes along with the food aversions…at eleven in the morning [I say] “we’re going to make this for dinner” but then at six o’clock I’m like, “I don’t want that, that doesn’t even sound good.” So knowing what I’m going to like, not even just like from day to day, but throughout the day has been hard. If we have something planned, my husband will make it and if I don’t eat any he’ll take the rest for lunch and I’ll find something that sounds a little bit better for me.*

*—Participant 5, age 26, first-time mom*

To a lesser extent, cravings also dictated food choices. Some craved simple foods, limiting variety and making it more difficult to cook nutritionally balanced meals, while others craved processed or convenience foods in place of home-cooked meals.

*I tried for so long to become vegetarian and I am…But I didn’t know I was pregnant until I started wanting hot dogs and hamburgers …and we don’t eat that…my husband has never had those kinds of things. And I’m like, what am I going to do? So we had two different meals going on in the house because I would want those things and then we have the normal things that we make for the family.*

*—Participant 17, age 35, mom of 2*

Fatigue was another common barrier to cooking, especially for participants with children already. Many women described feeling too tired to cook or just not being able to bring themselves to cook, this applied not only to the manual act of cooking but the mental energy required for planning meals. Participants anticipated that cooking would be equally if not more challenging postpartum because they would be exhausted from breastfeeding and busy taking care of baby.

*For me, it’s mostly fatigue. My son, he’s sunup to sundown he doesn’t stop, he’s always bouncing all over the place … me not being able to get as much sleep as I used [it is] difficult for me to cook as much as I used to… if I don’t have something that needs to go with that meal then I’ve got to go to the store, and if I don’t like going to the store then I can’t make that meal, and then I’ve got to make something different. At that point I’m over it, I don’t feel like cooking anymore, I’m going to order some food.*

*—Participant 1, age 30, mom of 1*

Due to their dietary limitations and family preferences, some participants cooked separate meals for themselves, their partners, and their children, adding extra layers of time, effort, and complexity to meal preparation.

*I have twin boys and they are on the smaller side so they require high fat meals whereas, I eat more vegetables since being pregnant. I can’t really eat meat. And then my husband is in the process of having all this dental surgery so then he has to have soft foods. So what I do is I just buy premade meals and put that in there for them. And then make something for myself. I have to do it easy.*

*—Participant 11, age 36, mom of 2*

In addition to the physiological challenges of pregnancy, participants found it mentally taxing to navigate extensive food and nutrition recommendations from health care providers, family, friends, and social media. Examples included reducing caffeine, limiting fish consumption, avoiding deli meats and unpasteurized milk products, and recommendations around breastfeeding and child feeding practices. Participants did not perceive most recommendations as absolute rules, and therefore constant exposure to new information entailed an iterative process of learning, evaluation, and prioritization. In other words, participants were not only constantly learning new recommendations, but also weighing the risks and benefits of adopting each recommendation.

*I think with it being the first, it makes it a little more intimidating trying to figure out what is actually necessary versus what’s just a good recommendation, especially like a lot of pregnancy books are like, “Oh, you’re only allowed to eat so many ounces of fish per week.” I’m like okay, do I really need to measure all of the fish that I eat? Is that necessary? I kind of gave up on trying to do all the specific things, I’m just trying to eat more vegetables and fruit. So it would be nice to have more guidelines as to what’s actually important versus what’s kind of important.*

*—Participant 6, age 24, first-time mom*

Navigating food and nutrition recommendations was also reported as challenging for women who had breastfed babies:

*I feel like it so much worse when you talk about breast-feeding … advice I see people giving …“you can’t have ginger. And you can’t have garlic. And you can’t eat anything spicy. And you can’t do this and you can’t do that.”...no wonder people feel like they can’t breast-feed. They feel like they have to not do any of these normal things which there’s no evidence of it being necessary.*

*—Participant 2, age 32, mom of 2*

Meanwhile, many associated food recommendations with judgement and pressure to “do everything right” regarding food and nutrition, and reporting that this added undue emotional stress.

*I was looking into more finger food for the early stages and found baby-led weaning, but there’s some baby-led weaning where it’s like, “Never give your kid purees! Never give them this!” And if you broke the rules already—me, at first when I was looking, I was like, “Oh, my gosh! I messed my kid up, because I gave him purees,” and then reading into it—it’s such a crazy topic with so many different opinions. I was like, “I’m just going to do what I want now.”*

*—Participant 12, age 28, mom of 1*

Despite this sentiment of feeling overwhelmed, participants were also dissatisfied with recommendations that were too generalized and superficial; they preferred more in-depth information so that they could better assess risk for themselves.

*…it’s about knowing actual risk for things. The risk of getting salmonella from cross-contamination of a cutting board that you cut raw chicken and then you chop your salad up that’s a serious risk. You can really get sick. The risk of you getting listeria from eating a turkey sandwich from Jimmy John’s is very low*

*—Participant 2, age 32, mom of 2*

Moms with previous pregnancies tended to be more confident and relaxed when it came to navigating food recommendations and coping with social pressure. Those with culinary, medical, or science training also expressed greater confidence in their ability to interpret and evaluate health recommendations.

*To an extent, there’s all this information about being a first-time mom and all the recommendations and sometimes they’re conflicting recommendations, and for the second, it just felt a lot easier, because we’ve been through this. I remember what helped, what didn’t and even though every pregnancy is a little bit different, it’s still not as much new information in every single day.*

*—Participant 7, age 34, mom of 1*

*So, the first time, you’re probably like, “Oh, my gosh, I can’t eat lunch meat.” And, now, I’m popping it in the microwave, just heat it up a little bit, then I throw on it a grilled cheese or something … some things, obviously, are there for a reason. Other things are merely suggestions, I guess.*

*—Participant 13, age 34, mom of 2*

Finally, pregnancy compounded many cooking challenges not specific to pregnancy. Many perceived time as a barrier to cooking, especially those who worked or had other children to care for.

*I love to cook but I feel like I don’t have time to cook and so I don’t know what to cook because I want to make this but it takes an hour, but my kid’s going to be screaming at me in 30 min, so what can I do? And my spouse and I both work and don’t get home…so it’s like it’s either in a crock pot, which is gross or it’s quick.*

*—Participant 15, age 36, mom of 1*

The constant responsibility of planning and cooking meals often led to burnout, regardless of whether participants enjoyed cooking. Repeating the same meals was boring but finding new recipes also required time and energy.

*I love cooking but I’m kind of staying home right now so I’m already cooking like three meals a day so it gets kind of tiring. And I have a little one, so I need to cook quickly, whatever it is.*

*—Participant 16, age 33, mom of 1*

*I cook because I have to eat and my husband hates to cook even more than I do. I don’t hate it. I don’t like it. It’s boring. And I’m not an imaginative person so my mental energy of figuring out what to cook kills me, so we eat out a lot.*

*—Participant 3, age 40, first-time mom*

Having to clean up afterwards also contributed to burnout and dissuaded participants from cooking.

*I hate cleaning up… I’ll take the easy way out with dinner not because I don’t feel like doing the effort of cooking a nice dinner but because I don’t feel like washing all the pots and pans afterwards.*

*—Participant 2, age 32, mom of 2*

#### 3.2.4. Cooking Education Program

In general, participants were interested in attending a cooking program during pregnancy, though many felt that it would be difficult to participate in a cooking program during the first trimester due to heightened nausea and aversions.

*Probably the second trimester [for a cooking class] because of the exhaustion and the food aversion. You don’t want that in a cooking class. Women are going to lay down or run to the bathroom, and then once you kind of get your second wind…you just kind of start planning for the rest of it once you start showing and everything.*

*—Participant 4, age 31, mom of 1*

However, others argued that it was important to engage women early in pregnancy for maximum benefits. They felt that education and support is generally lacking early in pregnancy.

*I couldn’t get in [for an appointment] for several weeks after I found out I was pregnant. And with this being my first pregnancy I was like, really?… But it would be nice if they sent even just an informational packet to you once you make the appointment and be like … here’s a list of things that you probably should stay away from.… Or just some instructions or information would be nice to have early on.*

*—Participant 10, age 30, first-time mom*

Participants agreed that a cooking program would be valuable during any pregnancy, though they would be more likely to attend during a first pregnancy when they were more motivated to learn and did not have to worry about childcare.

*I feel like you learn a lot of things in your first pregnancy and having your first kid that you use in the future, and I mean, you might not be opposed to going to a cooking class, but you might say, “Oh, well I don’t need that, and I don’t need to go, because I already know what to do because I just did it.”*

*—Participant 4, age 31, mom of 1*

*I think that every pregnancy is different. And then like, as you said, you can impart knowledge for those that have gone through it already. But their experience of pregnancy could be different this time around and they probably can learn something new as well.*

*—Participant 9, age 42, first-time mom*

Participants also thought it would be valuable to mix first-time moms and those with previous pregnancies who could share personal experiences.

*I think that having a mixture is good because you gain a lot of knowledge from people that have gone before you even if you don’t want to believe them and then you’re like goddammit, they were right.*

*—Participant 11, age 36, mom of 2*

The idea of a cohort model, where participants at roughly the same gestational age move through a series of classes together, was popular for enhanced social support and tailoring of content to the stages of pregnancy.

*I think it would be cool, too, kind of like in that you have your own group that you’re starting schooling with, you’re graduating with at the same time…I think that’s kind of cool because then you kind of established this bond and relationship with these people.*

*—Participant 9, age 42, first-time mom*

Participants discussed a broad range of content ideas, generally reflecting the conversation about motivators and barriers to cooking. Ideas included recipes, organizational strategies, culinary skills, nutrition, and food safety, with an overarching theme of social support, balance, and self-compassion. Participants agreed that recipes were an important component of a cooking program and were primarily interested in quick, easy recipes that use staple ingredients. They emphasized inclusion of budget-friendly and flexible recipes to maximize accessibility.

*For me, it’s less about the cooking skills because I feel like I do have a layperson’s knowledge of how to use my cooking equipment. It’s more of like getting a variety of recipes because, like I think I said I always get in a recipe rut and then I feel like frustrated and I can’t figure out anything because nothing sounds good. So just to have a resource that has a lot of different types of recipes would be really helpful.*

*—Participant 10, Age 30, first-time mom*

However, participants suggested providing multiple levels of difficulty or complexity for more advanced cooks or days when they felt more ambitious:

*I think it would be nice to have different levels of difficulty, because there’s a lot of nights where I may not want to cook but there’s some nights when I want to cook something fancy, so it would be nice to have different levels and have it laid out and say, well, this is for lazy nights, and this is a really fancy one.*

*—Participant 6, age 24, first-time mom*

Some articulated an interest in broader culinary skills that could be applied across a range of recipes. These ideas included knife skills, recipe substitutions, building flavor, how to cook vegetables and meat alternatives, and how to use herbs and spices.

*You might give people the recipes and talk through them, but it might not be a focus of the class…that way you’re focusing kind of on those basic skills …but telling people how they can be built on. So the skills to chop and onion …the skill is pretty basic, but once you have that skill, you can apply it across a lot wider range of cooking.*

*—Participant 7, age 34, mom of 1*

*I think something flexible would be nice that teaches you if garlic doesn’t sound good, maybe try this instead. Learning what actual different flavors do to what you’re cooking. Or if you don’t like chicken, try red meat or try whatever non-meat alternative might be, but learning the substitutions without being scared of messing up the whole recipe.*

*—Participant 12, age 28, mom of 1*

While participants already used a variety of organizational and meal planning strategies, they described this as a process of continuous improvement and were eager for new ideas. Examples included batch cooking, techniques for freezing food, and budgeting skills. In addition to weekly meal planning during pregnancy, participants emphasized planning for postpartum, for example, freezing meals ahead of time.

*I think what I would really want is help with the planning, organizing, meal prep that kind of stuff. That’s what I struggle with … being organized, so you have less of those days where, “oh my God, I’m exhausted. I don’t have anything ready. I don’t even know what I’m going to do. Let’s just get it hot and ready.” That’s what I feel like would be really beneficial to me, in general, in life but specifically with the pregnancy.*

*—Participant 3, age 40, first-time mom*

Some identified nutrition as an essential component of a cooking program. They were interested in learning about foods that promote the health of baby in utero and during breastfeeding as well as general dietary guidelines for macronutrients and calories.

*I definitely think at least adding in a section more on your breastfeeding would be really good, because I know your core intake has to be a lot higher when you’re breast feeding, and I don’t think a lot of people think about that.*

*—Participant 6, age 24, first-time mom*

Participants were also interested in dietary strategies for managing symptoms and preventing complications, for example, healthy snack ideas and mindful eating strategies to manage cravings and prevent excessive weight gain or dietary strategies to combat nausea and aversions.

Content about cooking for baby was a lower priority for most participants. Many planned to breastfeed and therefore were not immediately concerned about what they would feed their infant. Participants planned to give the baby the same food they cooked for themselves (i.e., baby led weaning), modify simple foods or foods they cooked for themselves (i.e., mashing or pureeing), or use store-bought baby food. Many perceived “cooking” meals for baby as unnecessary and participants were concerned that elaborate baby food recipes would add to the pressure to be “supermom”.

General child feeding guidelines were deemed important content, including approaches to weaning, when to introduce specific foods (i.e., honey, peanut butter), use of salt, sugar, and seasonings in baby foods, and safety tips to avoid choking hazards. However, participants felt that the focus should remain on mom and pregnancy.

*I’ve always thought making your own baby food would be nice but I’ve never done it before… I don’t know what to add to it and what part of the vegetable.... I don’t know if that would be a thing for a cooking class with pregnancy or not, because I mean you kind of want to prepare for it in the long run.*

*—Participant 4, age 31, mom of 1*

Participants urged intentional presentation of nutrition and food safety information, warning that too much information would be overwhelming while too little information would come across as over-generalized. Participants also valued knowing the rationale for nutrition and food safety recommendations so that they could assess risk for themselves.

*[The recommendations] can definitely be overwhelming…there’s a lot of, “don’t eat these things”, but not like, “here’s a good breakfast that you could have”…I feel like if there was more examples of what to eat I think that would be helpful.*

*—Participant 5, age 26, first-time mom*

*I think it would be helpful to allow us to make our own decisions if they’d tell us what the possible repercussions are instead of saying, oh, you’re only supposed to have this if this, but then they don’t tell you what happens. Is it something that, oh, you might not feel well, or is it going to potentially harm your baby?*

*—Participant 6, age 24, first-time mom*

Participants consistently identified peer support and positive messaging as essential aspects of any program for pregnant women. Women thought it would be beneficial to share ideas and experiences with other moms, even brainstorming how social support could be included in a virtual cooking program through an online discussion forum. They emphasized the use of positive messaging to promote balance and self-compassion, for example, using flexible recipes and providing a list of strategies to try or foods to eat rather than only a list of foods to avoid.

*Initially when I was diagnosed with pre-diabetes they offered a nutritional program at [my hospital] … and they had cooking classes … that would be nice to do early on in the pregnancy [then] you’re in a room full of women that are kind of in the same boat as you so it’s nice to relate rather than that program of nutrition was just very blah… you get like, “Here’s what not to eat” but then they can’t relate you.*

*—Participant 9, age 42, first-time mom*

*I think an important aspect would be to counteract the toxic culture we live in that expects every mom to be supermom. I think that if we had a program that says what you’re doing is great. Continue doing those things. Here’s some things that may help you. You don’t have to go all out but if you want to there are resources.*

*—Participant 3, age 40, first-time mom*

In terms of program format, most participants preferred an in-person, hands-on class over an exclusively virtual program. However, they thought that an online program would be more feasible, especially during the first trimester or for those with children.

*Online would definitely be easier if you already have kids, or the option of having the childcare, but I would like a group setting. It’s more fun for me. I’m more of a social person, so I like group settings more.*

*—Participant 8, age 27, mom of 1*

Participants were most in favor of a hybrid format that incorporated both in-person and online learning, for example, reviewing content on their own schedule at home then meeting in-person once a month or once per trimester.

*I think kind of a combination approach would be the most attractive. So say it’s a ten-week thing, have … weeks where it’s an in person social setting, and then you’ve got recipes and tools that come through once a week in between those in-person sessions that build on the skills that were taught in the session. So you’ve seen it demonstrated, you had a chance to practice hands on, but then you can take it and practice those skills [at home].*

*—Participant 7, age 34, mom of 1*

For an in-person component, participants suggested demonstrations, samples, and hands-on cooking, because they thought these strategies would increase their likelihood of using the information at home.

*I think having samples and demos and advertising that, too, would probably (1), get people interested in coming, and (2) maybe get them to actually implement the things to try them at home a little bit more. If you taste something there and it’s delicious you’re probably more inclined to do it yourself than if somebody just tells you about it.*

*—Participant 2, age 32, mom of 2*

Regardless of the primary instructional format, participants wanted to be able to access educational materials, such as videos or handouts, online. Some envisioned an online discussion platform to enhance social support. Participants also said they would be more likely to attend a program at the same location as other prenatal services (i.e., the hospital or clinic) and noted the importance of accessibility by public transportation. Participants also would be more likely to attend a program if they were referred by their health care provider.

*I think both would be nice to meet once a month with a group of women that are looking for the same thing. And talk about everything. And then also be able to access those resources online especially if you guys are going to provide substitutes or suggestions for recipes because I just think that’s easier [than getting paper] and more environmentally friendly.*

*—Participant 11, age 36, mom of 2*

Participants had mixed opinions about whether partners should be included in the cooking program. Those not in favor of including partners were not in a stable relationship with the baby’s father or argued that their partner would not want to attend or would not enjoy the program. Participants were also concerned that including partners would make it awkward or reduce female peer support. Some explained that having to “babysit” their spouse would take away from their own enjoyment of the program. One group suggested providing a separate education program or “support group” for fathers or fathers to be.

*I think it adds an additional layer of complexity that I may not be able to handle only because I don’t want to have to kind of babysit and be like, okay, we’ve got to go to this thing.*

*—Participant 9, age 42, first-time mom*

Those in favor of including partners thought that their partner would enjoy the program or that it would be a fun activity to do together. Some participants felt that it was important for their partner to learn nutrition and cooking information so that they could better support them through pregnancy.

*Having your partner on board is really, really important… especially during your first trimester…Having them have that understanding of what the recommendations are or having easy go-to meals…not just targeting me as the primary cook…but having it be a whole family…we’re going to get everybody on the same page. We’re going to give you all the same recommendations so that…your partner has those tools.*

*—Participant 7, age 34, mom of 1*

Finally, in addition to highlighting the need for childcare, participants discussed pros and cons of having children participate in the program. They were generally interested in cooking with their children at home and thought that it was important to cook with their children. However, allowing children to participate seemed logistically challenging and some feared that it would detract from the pregnancy focus and be unfair to first-time moms.

## 4. Discussion

In this formative qualitative study, we explored cooking behavior during pregnancy independent of broader lifestyle intervention, as well as participant preferences for a stand-alone cooking skills intervention during pregnancy. The findings demonstrate interest in cooking education during pregnancy and characterize motivators, strategies, barriers, and learning needs related to cooking during pregnancy and postpartum. In this study we intentionally did not define cooking or ‘healthy cooking’ for participants; rather, we discussed cooking at home generally and left definitions open to interpretation. Participants perceived home cooking to be an important strategy for maintaining a healthy diet during pregnancy. While cooking at home more frequently is associated with better diet quality, not all home-cooked meals are healthy and perceptions of ‘healthy cooking’ vary [17]. Common motivators for cooking at home during pregnancy included needing to feed other children, promoting fetal growth and development, preventing foodborne illness, preventing complications, and managing pre-existing health conditions. Common strategies for cooking healthy meals at home included meal planning, buying in bulk, using whole, unprocessed ingredients, and limiting added salt, fat, and sugar. Common barriers included pregnancy symptoms, overwhelming nutrition recommendations, catering to family preferences, time, and burnout. Finally, content/format preferences and learning needs for a cooking program included organizational strategies, recipes, basic nutrition information, social support, and accessibility.

Pregnancy and the first 1000 days of life are a critical period during which nutrition can have lifelong impacts on mother and child [1,8,9]. However, pregnancy is also a life stage that presents particular challenges to preparing and consuming healthy meals and snacks [20,21]. Our findings on barriers to cooking during pregnancy, such as lack of time, burnout, and catering to family preferences, are not specific to pregnancy and are largely consistent with prior literature on challenges to cooking in the general population [31,32]. Prior studies in low-income populations have identified food affordability and access as key barriers to home cooking [33,34,35]; while these were not salient themes in this study, participants acknowledged that cost and access are likely barriers for other people in their communities. Pregnancy-specific challenges to cooking were consistent with prior literature on barriers to adopting nutrition and physical activity recommendations during pregnancy [8,20,21,36,37]. Pregnancy symptoms and “coping with their own physical changes during pregnancy” make it difficult to follow through despite strong intentions around health behaviors [8,21]. Similar to our findings, previous qualitative studies have found that women feel overwhelmed by food recommendations during pregnancy, which they perceive to be constantly changing and sometimes conflicting, making it difficult to operationalize dietary guidelines into tangible health behaviors [20,21]. Cooking interventions—especially hands-on programs—are well suited to addressing some of these challenges by providing tangible behavioral strategies for implementing food and nutrition recommendations and providing an opportunity to explain the research supporting these recommendations.

Findings related to interpersonal factors and household cooking dynamics varied somewhat from previous studies. Nearly all participants in this study described being the primary cook in their household and did not express strong opinions surrounding partner involvement in cooking, perhaps because most participants accepted their role and/or felt supported by their partners and/or other family members in meeting their dietary goals. In a previous focus group study of primarily non-Hispanic black pregnant women, the majority of participants were not the primary cooks in their households and described lack of social support from family members as a barrier to achieving their dietary goals [37]. While previous literature emphasizes involvement of partners as a key opportunity in prenatal interventions [14,21], other family members (i.e., parents or siblings) may be equally or more important support people when it comes to cooking. When considering this in the context of cooking program planning, participants noted that inclusion of partners may reduce female peer support and exclude single mothers. These trade-offs should be considered in the development of future cooking interventions, perhaps including not only partners but other social support people.

In terms of motivation for food- and nutrition-related behavior change during pregnancy, perceptions around gestational weight gain and the importance of prenatal nutrition are variable [38,39,40,41]. Mothers in this study were generally concerned about excessive weight gain and identified home cooking as tool for weight management. However, some also expressed attitudes of wanting to “enjoy pregnancy and not worry too much”, an attitude that has also emerged in previous qualitative studies [20,39]. Prior qualitative studies have found that mothers’ perceptions surrounding appropriate gestational weight gain often do not align with clinical guidelines or that they do not associate excessive gestational weight gain with health risks [37,40]. Furthermore, health care practitioners report feeling uncomfortable and ineffective in addressing gestational weight gain [37,41] and it is unclear to what extent practitioners are addressing maternal diet and weight management in routine prenatal care [42]. Participants in this study expressed frustration with lack of nutrition education in routine prenatal care and desired a more preventative rather than reactionary approaches to potential nutrition-related complications. Cooking education interventions offer an opportunity to bridge this gap in prenatal care in a way that is accessible and culturally relevant to participants with diverse backgrounds and perceptions about nutrition. Though not explicitly discussed here, preconception cooking skills interventions may also be more convenient for participants and would be an opportunity to build key skills that would carry through pregnancy and beyond.

Several cooking skills interventions targeting pregnant and postpartum women already exist. A systematic review of culinary nutrition education interventions during preconception, pregnancy, and postpartum, found that the majority (80%) of interventions were “conducted during the postpartum period and targeted nutrition issues in infants and young children”; only 20% of interventions were conducted during pregnancy and none pre-conception [14]. Cooking programs conducted during pregnancy tend to commence during the second trimester [43,44,45,46,47,48]. Participants in our study thought it would be important for an intervention to start earlier in pregnancy; they expressed frustration with lack of early prenatal care and thought that a cooking program could help address first-trimester symptoms such as food aversions, nausea, and fatigue. However, they concurred that it would be most feasible to attend a program during the second trimester. Incorporating both in-person and online learning could be an effective way to address these challenges.

The modality and content of existing cooking interventions targeting pregnancy are quite variable, with the majority of cooking programs delivered as one component of a larger lifestyle intervention [43,44,45,46,47,48]. Most existing programs have used primarily in-person, hands-on learning in a group setting [43,44,47,48], some used demonstrations only [45] (no hands-on component) or one-on-one home visits [46]. Though participants in our study tended to prefer hands-on, in-person learning, they also suggested a “hybrid” program that incorporates both hands-on and online learning (including videos, recipes, and/or an online discussion platform) for increased convenience and accessibility. Indeed, participant retention and program attendance seems to be a common issue for exclusively in-person prenatal cooking interventions, no matter the stage of pregnancy [44,47,48]. Though research on hybrid programs specifically is quite limited, one randomized controlled trial of a dietary intervention during pregnancy that included an in-person cooking class along with “access to a password-protected website with recipes and practical tips on cooking” was feasible and resulted in favorable dietary outcomes [43].

Participants in our study strongly preferred a group setting for enhanced peer social support, consistent with findings from numerous exploratory studies on prenatal nutrition learning needs [21,37,49]. Peer social support has also been documented as an important interventional strategy [44,47,48] as well as a key secondary outcome in cooking interventions [32,38]. In one qualitative study assessing three year outcomes of a group prenatal care program that included recipes, cooking information, and food samples, participants reported that peer social support “facilitated learning” and allowed for more in-depth exploration of cultural factors that affect food and health choices [38]. Another cohort-style intervention that included seven group cooking classes noted peer social support as a potential facilitator to behavior change and improved mental health [44]. A pilot evaluation of an intervention (with a cooking component) targeting pregnant teenagers “encouraged discussions about food and nutrition and any pregnancy problems” found that participants strongly favored the “relaxed atmosphere” and the inclusion of only teenage females, which may have augmented sense of peer social support [48]. Further work is needed to determine how to effectively facilitate peer social support in virtual or hybrid programs.

Existing cooking interventions during pregnancy incorporate a range of content, though in-depth descriptions of content are often absent from the literature, which makes it challenging to determine to what extent the content preferences and learning needs expressed by participants in the present study are being met by existing programs [14]. Existing programs most frequently include nutrition guidelines, food safety information, and recipes, and sometimes content related to grocery shopping and infant feeding [43,44,45,46,47,48]. While participants in this study felt that nutrition and food safety were important, they also warned that these topics could be more overwhelming than helpful. In general, cooking skills interventions may inherently help to address this issue by focusing on behavioral strategies that seamlessly integrate nutrition and food safety messaging with cooking and food skills that build food agency and self-efficacy around food choices and cooking.

One particularly interesting insight from our study was the focus on organizational skills such as meal planning, batch cooking, and budgeting to help reduce the mental toll of meal planning and management of household food resources. Similarly, in a recent qualitative study, mothers of children under 18 years old identified planning and resourcefulness as key food skills for consuming a healthy diet [50]. Participants’ interest in simple recipes also speaks to a need for tools to help reduce the “mental energy” of meal provision. Two existing interventions conducted during pregnancy included content about organization, time management, and budgeting skills (though it was unclear to what extent this content was presented in the context of meal provisioning) [47] and meal planning [46]. However, neither of these studies assessed outcomes related to cooking or diet quality. A growing body of literature supports a broader conceptualization of cooking skills to include the “complex set of deliberate procurement, budgeting, organizational, conceptual, and decision-making skills” that are “specific to an individual’s context and environment” [51]. Additional content themes not currently represented in the literature included meal planning for postpartum, basic culinary skills, and basic child feeding guidelines (in addition to infant feeding guidelines). Collectively, these findings support several recommendations for future cooking interventions during pregnancy: greater emphasis on organization and planning skills related to cooking, mindful inclusion of nutrition and food safety content to avoid information overload, and greater emphasis on basic recipes and culinary skills that can be applied across a broad range of ingredients.

Unlike prior qualitative, formative studies [8,20,32,37,39,52] exploring cooking skills interventions and prenatal/postpartum nutrition and weight management interventions, we did not observe strong preferences for culturally-tailored content. This is not surprising given that our groups did not represent any one cultural group and were quite heterogenous in terms of race and income compared to previous studies [8,32,37,39,52]. Interestingly, relatively few studies of cooking interventions during pregnancy have explicitly highlighted culturally-tailored approaches. One pilot prenatal nutrition intervention with a cooking component, targeting Latina women, emphasized “family values, traditions, and experiences…to connect desired behaviors with cultural norms and values, and highlight cultural strengths to support behavior change” [47]. Future research should explore the role of culturally-tailored content in cooking interventions during preconception, pregnancy, and/or postpartum.

This study has several strengths. To our knowledge, it is the first formative, qualitative study to examine cooking behavior and learning needs during pregnancy, outside of the context of general lifestyle interventions. In particular, by using focus groups, we were able to engage participants in rich discussion that strengthened our understanding of the topics covered in the focus groups. Additionally, this study followed the COREQ guidelines for implementing and reporting qualitative research, demonstrating rigor in data collection and analysis. This study also has several limitations. Due to selection bias, women who already enjoyed cooking and/or felt confident in their cooking skills were likely disproportionately represented in this study. Additionally, focus groups included only English speakers, limiting the sociodemographic diversity of the study population and excluding groups who may be especially vulnerable to suboptimal nutritional status during pregnancy. For example, previous qualitative studies found greater emphasis on food cost, food access, and culturally-appropriate education, whereas these were not major themes in this study. Furthermore, this study was not designed to investigate differences in perspectives based on age, race/ethnicity or other socioeconomic or demographic factors, though there may be meaningful differences that would be important to understand to create tailored interventions for different populations. These subjects would be worthwhile topics for future research and should be explored during preliminary phases of any intervention development [53]. Furthermore, in this study we did not define cooking or ‘healthy cooking’ for participants; rather, we discussed cooking at home generally and left definitions open to interpretation. Not all home cooking is healthy, and evidence shows that people define cooking very differently, with some definitions including pre-prepared and highly processed foods [33]. Finally, though generalizability is not a goal of qualitative research, our results should be considered within the specific context in which the data were collected; namely, from a small sample of women from a single geographic area.

## 5. Conclusions

Nutritional status during the first 1000 days of life, including pregnancy, is critical for the long-term health of mother and child. Pregnancy is a life event that can be a strong cue to action that facilitates a greater focus on nutrition and healthy eating [54]. However, pregnancy also presents many unique challenges to adopting nutrition recommendations, such as preparing and consuming healthy meals. This study identified a range of motivators, strategies, challenges, learning needs, and program preferences related to healthy cooking during pregnancy, with implications for the development of future cooking skills interventions targeting pregnant women. Our findings support the development of cooking skills interventions for pregnant women as a strategy for improving maternal and child nutrition and provide rich data to inform program development.

## Figures and Tables

**Table 1:** Demographic characteristics of focus group participants.

	All Focus Group Participants (*n* = 20)	
Age (years)		
Mean (SD)	32.1 (5.6)	
Gestational age (weeks)		
Mean (SD)	18.3 (9.6)	
Number of children		
Mean (SD)	1.4 (0.5)	
Age of children (years) ^a^		
Mean (SD)	5.5 (3.8)	
	*n*	%
Has children	14	70
Education		
High school graduate	2	10
Some college	2	10
College graduate	16	80
Race		
Black	3	15
Hispanic	3	15
White	12	60
Asian	2	10
Other	0	0
Marital status		
Single	2	10
Married	16	80
Living with a partner	1	5
Divorced/separated/widowed	1	5
Employed		
Part time	8	40
Full time	9	45
Not working/retired	3	15
Pre-pregnancy weight status		
Normal weight	8	40
Overweight	9	45
>20 lb overweight	3	15
Cooks dinner ≥ 5 nights/week	14	70
Has a working stove	20	100
Has a working oven	20	100
Has a working fridge	20	100
Has a working freezer	18	90
Has a working microwave	19	95

^a^ among participants with children.

**Table 2:** Key Focus Group Themes and Sub-Themes.

Themes	Sub-Themes
Motivators to home cooking during pregnancy	
Attitudes about cooking	Cooking is enjoyable Home-cooked food is perceived to be healthier
Feeding other children	Need to cook healthy meals for my other children Want to set a good example for my other children
Cooking and pregnancy	Avoiding foodborne illness Avoiding pregnancy complications Managing pre-existing health conditions Promoting health of baby during pregnancy and breastfeeding Prior pregnancy loss or infertility makes me more cautious about food Multigravida women felt more confident and relaxed about food recommendations and social pressure
Cooking strategies and household dynamics	
Household cooking dynamics	I am the primary cook in my household I am responsible for planning meals even when I am not cooking Spouse/partner or other family member helps with cooking Spouse/partner support is important Little/no change to household cooking dynamics during pregnancy
Perceptions of healthy cooking	Focus on balance and variety of food groups Knowing what is in my food or eating whole foods Limiting calories, sodium, and/or carbohydrate
Strategies for healthy cooking	Meal planning Using leftovers Buying and/or cooking in bulk and freezing Reading nutrition labels
Barriers to cooking during pregnancy	
Pregnancy- specific barriers	Pregnancy symptoms (nausea, fatigue, cravings, aversions, etc.) Food and nutrition recommendations are overwhelming Peer/societal pressure to be “supermom” Every pregnancy is different Breastfeeding/having a newborn is more challenging than pregnancy
General barriers	Catering to family preferences and dietary restrictions Mental energy of meal planning Time and clean-up
Ideas for a Cooking Education Program	
Logistical considerations	Begin during first trimester but challenging to attend in-person cooking program during first trimester Valuable during any pregnancy (i.e., first vs. second) Cohort model Hybrid format with both virtual and in-person, hands-on components Accessible location Program associated with health care provider is more credible Provide childcare
Including partners, children, or other family	Including partners would make it awkward/reduce peer support Partner would not enjoy/don’t want to ‘babysit’ partner Partner or other adult family member would benefit from information Pros and cons to including children
Cooking content	Meal planning strategies and organizational skills Recipes: quick, simple, basic ingredients, freezer-friendly, affordable Include multiple levels of difficulty Culinary skills: herbs and spices, building flavor, recipe substitutions, knife skills, ‘making vegetables taste good’ Food safety during pregnancy
Nutrition content	Managing pregnancy symptoms (cravings, aversions, nausea, etc.) Nutrition to support baby in utero Nutrition to support lactation General child feeding guidelines, but not cooking for baby
Peer support	Peer social support is important Online discussion platform
Positive messaging	Focus on balance and self-compassion Reduce pressure to ‘do everything right’ regarding food Keep it simple: avoid overwhelming nutrition recommendations

## Appendix A. Focus Group Question Guide

Question Number	Question	Prompts	Aim
1	Icebreaker: To get started, can we go around the table and everyone say their name and a favorite meal you like to cook (or, if you don’t like to cook, a favorite meal you like to eat)	Remind people to say their name, and keep it brief	Get people talking, allow the transcriber to recognize names/voices
Great, thank you. From now on, we won’t go around the room for each answer. But we’ll try to have more of an informal conversation. So, feel free to respond to what other people are saying			
2	Let’s start by talking about how everyone feels about cooking?	If no one speaks up… Does anyone particularly enjoy cooking? Does anyone not like cooking much?	Gain insight into participants thoughts around cooking
As I mentioned earlier, we are interested in knowing more about your thoughts on cooking and how that relates to health, particularly in the context of pregnancy. The next few questions will relate to your cooking knowledge and skills and how becoming pregnant may have changed the way you feel about this.			
3	Is there anything that encourages you or discourages you to cook now since becoming pregnant? Are there any challenges you face, now that you are pregnant when it comes to cooking?) - What do you find most challenging about cooking/preparing foods now that you are pregnant)? How do you think you will feel about cooking and preparing meals once your baby arrives, and then 6–12 months down the road?	Desire to be healthier, consuming the right foods Morning sickness, fatigue Ask participants to describe these challenges Are there things that have motivated you to cook now that you are pregnant? Interested, excited, overwhelmed Do you have any particular concerns about cooking and preparing meals once you have had your baby?	What are the challenges (especially those caused by pregnancy) Establish the concerns, challenges participants feel/perceive they have after the baby is born
4	I would now like to focus on how you think cooking relates to health. Does anyone have any thoughts or comments around health and cooking? Based on what we have just discussed, have your thoughts about cooking and health changed since you has been pregnant?	What does healthy cooking mean to you? Is cooking and health important to you? What issues are you concerned about (good nutrition, gestational diabetes, weight, etc.)? Has your pregnancy changed your relationship with cooking? Has it changed your thoughts on health? Have you had any particular food safety questions or concerns related to cooking/eating during pregnancy? What about for immediately following pregnancy?	Gather participants opinions on cooking Is it something participants find important? What is the impact of pregnancy on this?
We would like to know a little bit more about who else in your household cooks. Please keep this in mind when thinking about the following questions. What are the dynamics of cooking in your household? Has that changed with pregnancy and will it change with a baby? And what would be the best way to incorporate that into a cooking program to accommodate this?			
5	What are the dynamics of cooking in your household? Who does what? Has this changed with pregnancy? Do you think this will change with a baby? What is the best way to take these dynamics into consideration when developing a cooking program?		Who else cooks in the household Do you share the cooking Has this changed since becoming pregnant, with a baby, etc.? Should the cooking program include (or not include) partners
We are hoping to develop a cooking program for pregnant women. In the next few questions we are going to ask what you would like/not like in a cooking program			
6	If we offered a cooking program to pregnant women, what do you think would appeal most to pregnant women, in terms of the stage of pregnancy, how to pitch it, etc.? Would you attend?	Why? Why not? When would be the preferred time to participate (i.e., what stage of pregnancy)?	Would participants use a cooking program? What stage of pregnancy would they be most likely to attend?
7	If we offered a cooking program to pregnant women, what would you expect the program to include? Consider what you want to know during pregnancy What you want to know with a baby? What would you expect the program NOT to include?	Any particular topics (e.g., food based, e.g., pasta; cuisine based, e.g., Italian; equipment based, e.g., slow cooker or BBQ); meal preparation or planning (e.g., finding recipes, preparing shopping lists, budgeting); food safety; nutrition; cooking tips for new mothers, cooking with a baby, cooking for a baby? What do you think would be most helpful/needed for you or other women in your social network/community?	
9	How would you prefer the cooking program to be delivered?	1. Group program face to face. 2. Group but over internet. 3. Individual over internet. 4. With partners or without. 5. With pregnant women only or including those with babies?	
9	What would help you to participate in a cooking program while pregnant or shortly after your baby is born? What barriers, if any, would make it difficult to participate in a cooking program, while pregnant or shortly after your baby is born?	1. Location 2. Time 3. Childcare (if >1 child) 4. Pregnancy itself (e.g., morning sickness)	
10	As we wrap up, is there anything else you’d like to add that we didn’t cover?	Needs/wants in a program? Changes regarding food/cooking and pregnancy? Things you think are important for us to know that we didn’t touch on?	
